# Does the Fear of COVID-19 Impact on Menopausal Symptoms in Women? A Research Investigation

**DOI:** 10.3390/jcm13154576

**Published:** 2024-08-05

**Authors:** Nurseli Soylu Erener, Salime Mucuk, Fulya Çağlı

**Affiliations:** 1 Department of Gynecology and Obstetrics Nursing, Faculty of Health Sciences, Erciyes University, Kayseri 38280, Türkiye; smucuk@erciyes.edu.tr; 2 Department of Obstetrics and Gynecology, Faculty of Medicine, Erciyes University, Kayseri 38280, Türkiye; f.cagli@yahoo.com

**Keywords:** menopause, COVID-19, fear, SARS-CoV-2

## Abstract

**Objectives:** This study aimed to determine the relationship between the fear of COVID-19 and menopausal symptoms in women during the menopausal period. **Methods:** This study is a descriptive cross-sectional study. This study was completed with a total of 161 women who met the inclusion criteria. Data were collected face-to-face using the Personnel Information Form, Menopause Symptom Assessment Scale, and Coronavirus 19 Phobia Scale. **Results:** There is a slightly positive relationship between the social subscale of the COVID-19 fear scale and the somatic subscale of the menopause symptoms scale. There is a slightly positive relationship between the economic subscale of the COVID-19 scale and the psychological subscale of the menopausal symptoms scale. There is a slightly positive correlation between the total score of the total COVID-19 phobia scale and the somatic and psychological subscales of the menopause symptoms assessment scale. However, there is no significant relationship between the total score of the COVID-19 phobia scale and the total score and urological subscale of the menopause symptoms assessment scale. **Conclusions:** It has been determined that fear of COVID-19 is slightly positively associated with somatic and psychological complaints in menopausal women.

## 1. Introduction

The menopausal period marks a significant transition in a woman’s life [1,2], and it is addressed explicitly due to its effects on women. With the depletion of follicles in the ovaries, ovarian function ceases [3]. The World Health Organization (WHO) defines menopause as the ‘permanent cessation of menstruation due to loss of ovarian activity’ [4]. For many women, this transition can be liberating, as it marks the end of fears of pregnancy and the cessation of dysmenorrhea. However, for some women, it can also carry negative connotations associated with aging [1].

Due to the decrease in estrogen levels in women during the menopausal period, some physical and psychological changes occur, leading to various issues [1,5,6]. Hot flashes, night sweats, sleep disturbances, fatigue, depression, brain fog, decreased libido, increased anxiety [1,7], headaches, dizziness, palpitations, nausea, anxiety, irritability, loss of appetite, vaginal dryness, dyspepsia, urethral syndrome, dry skin, and nail brittleness are among the problems that can be experienced during the menopausal period [5,8]. Approximately one-third of women experience the symptoms associated with menopause. The symptoms associated with menopause can be extremely distressing and can significantly impact various aspects of women’s lives, including personal, social, and professional areas [9]. A systematic review has determined that psychological symptoms experienced by women during the menopausal period include anxiety, panic attacks, concentration difficulties, irritability, depression, forgetfulness, fatigue, dizziness, dissatisfaction with life, and emotional fluctuations [10].

The coronavirus disease 2019 (COVID-19) virus first emerged in December 2019 in the city of Wuhan, China, and was declared a pandemic by the World Health Organization (WHO) on 11 March 2020 [11]. Starting as a regional health problem, COVID-19 quickly spread worldwide, becoming a pandemic [12]. During epidemic outbreaks, individuals often experience various psychological disorders such as fear, panic, or anxiety [13]. It is stated that the COVID-19 pandemic has led to many psychological effects such as stress, depression, anxiety, and fear [14,15,16,17,18]. Similar epidemics such as influenza A (H1N1), severe acute respiratory syndrome (SARS), Middle East respiratory syndrome (MERS), Ebola, and Zika have been reported to impact mental health negatively, leading to fear and anxiety disorders [19,20,21,22,23].

Decreased physical activity, changes in diet, and depression due to COVID-19 quarantine are expressed as important risk factors that cause postmenopausal women to experience more menopausal symptoms [24]. The unpredictability of the COVID-19 situation and the uncertainty of the end of the pandemic, the lack of certainty regarding treatment, and the high rate of infection and mortality are the most important factors that cause individuals to experience anxiety. Therefore, COVID-19 affects people’s mental health at individual, interpersonal, and social levels, as well as physical health. An important feature of infectious diseases is that they cause individuals to experience fear. Fear is directly related to the rate of infection and mortality of the disease. The COVID-19 virus has caused individuals to experience more fear due to its relatively high incidence and mortality rate [25].

In women during the menopausal period, symptoms of anxiety disorders can be observed [26,27]. When the literature is reviewed, it is found that anxiety levels increase during the menopausal period [28,29,30], and a positive relationship between menopausal symptoms and anxiety has been identified [31].

Considering these studies, it has been hypothesized that there may be a positive relationship between the fear of COVID-19 and menopausal symptoms. At the same time, there are studies examining the fears and anxieties of pregnant women [32,33,34], women in the postpartum period [35,36], women undergoing cancer treatment [37,38,39], and women applying for gynecological screening tests [40,41] during the COVID-19 pandemic. However, no study has been found that specifically investigates the relationship between the fear of COVID-19 and menopausal symptoms in women. Therefore, this study aimed to determine the relationship between the fear of COVID-19 and menopausal symptoms in women during the menopausal period and contribute to filling this gap in the literature.

## 2. Method

### 2.1. Study Design and Participants

This study is descriptively designed to determine the relationship between the fear of COVID-19 and menopausal symptoms among women in the menopausal period. The study population consisted of women who applied to the Gynecology Outpatient Clinic of a university in the Central Anatolia Region of Türkiye between August 2021 and July 2022 and volunteered to participate in the research. A total of 668 women were interviewed during the specified dates. Among them, 248 women had chronic diseases (such as diabetes, hypertension, heart disease), 49 women had undergone surgical menopause, and 210 women had contracted COVID-19. Therefore, they were not included in the study. According to the hospital’s procedure, since not every woman visiting the outpatient clinic undergoes a COVID-19 test, they were asked about symptoms of COVID-19 infection (such as fever, cough, difficulty breathing, sore throat). The women had been vaccinated against COVID-19 previously. A total of 161 women were included in the research. The researcher collected data through face-to-face interviews.

Women who had been in menopause for at least one year, had not had COVID-19, were not undergoing hormone replacement therapy, and were willing to participate in the study were included. Women who had undergone surgical menopause, had chronic diseases (diabetes, hypertension, asthma, chronic obstructive pulmonary disease), or had psychiatric diseases (bipolar disorder, depression, obsessive-compulsive disorder) were excluded from the study.

### 2.2. Measures

#### 2.2.1. Personnel Informational Form

The Personnel Informational Form survey consists of 21 questions covering sociodemographic characteristics and gynecological and obstetric information, as well as statements related to the COVID-19 pandemic.

#### 2.2.2. The Menopause Symptom Assessment Scale (MSAS)

The Menopause Symptom Assessment Scale (MSAS) was developed in German in 1992 by Schneider et al. (2000) to measure the severity of menopausal symptoms, and it was adapted into English in 1996 [42,43,44,45]. It was adapted into Turkish by Gürkan in 2005 [46]. The scale consists of 11 items and is of the 5-point Likert type. It has three subscales: somatic, psychological, and urogenital complaints. The scale’s total score is calculated based on the scores given for each item. The lowest possible score from the scale is 0, while the highest score is 44. An increase in the total score from the scale indicates an increase in the severity of symptoms experienced. The total Cronbach’s alpha reliability coefficient for the MSAS is 0.84. For the subscales, the Cronbach’s alpha values are 0.65 for somatic symptoms, 0.79 for psychological symptoms, and 0.72 for urogenital symptoms [46].

#### 2.2.3. The Coronavirus 19 Phobia Scale (C19P-S)

The Coronavirus 19 Phobia Scale (C19P-S) is a self-assessment scale developed to measure the phobia developed against the coronavirus. It is a 5-point Likert-type scale. The scale items are evaluated on a scale of 1–5 points. The scale consists of four subscales: psychological, somatic, social, and economic. Subscale scores are obtained by summing the scores of the items related to that subscale, while the total C19P-S score is obtained by summing the subscale scores. The score that can be obtained from the scale varies between 20 and 100 points. Higher scores indicate an increase in both the subscale and overall fear of coronavirus. The Cronbach’s alpha reliability coefficient for the internal reliability of C19P-S is 0.925, and for internal validity, it is 0.926. The internal consistency coefficients for the subscales range between 0.85 and 0.90 [13]. 

### 2.3. Statistical Analysis

In this section, we report the statistical analysis results. Our data have *n* = 161 observations, including the personal attributes and responses of the scales. First, we detected the potential outliers using Mahalanobis distance with the deterministic minimum covariance estimator [47] and discarded some observations out of the critical bound.

After the data preprocessing, descriptive statistics, normality test results, and reliability findings were provided. The normality of the scale scores was checked using a Shapiro–Wilk’s normality test, and the reliability was evaluated using Cronbach’s alpha coefficients. The reliability coefficients for each scale were obtained via psych package [48]. Besides the normality tests, we provided histograms with the ggplot2 package [49].

All the statistical implementations were performed via R software 4.3.1. [50]. We considered the error level as α = 0.05 for the statistical interpretations. A Spearman correlation analysis was used, and the scatter plot was given to represent the relationships using the ggcorrplot package [51]. Since the normality does not hold for the scale scores (i.e., responses), we conducted a quantile regression analysis [52]. In the regression part, we considered the subscales of the menopausal symptom assessment scale as dependent variables. We presented the regression findings benefiting data visualization methods via the sjPlot package [53].

### 2.4. Ethics

This study has been approved by the Ethics Committee of a university’s Clinical Practice (approval no: 2021/530; approval date: 28 July 2021), and institutional permission has been obtained from the institution where the study was conducted. Women to be included in the research were provided with an explanation of the purpose of the study and signed an informed consent form.

## 3. Results

**Figure 1:**
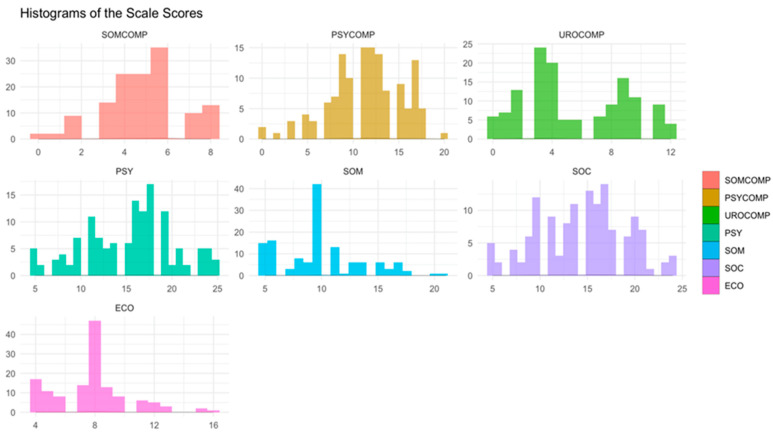
Histogram of the scale scores. SOMCOMP: somatic subscale of the Menopause Symptom Assessment Scale, PSYCOMP: psychological subscale of the Menopause Symptom Assessment Scale, UROCOMP: urogenital subscale of the Menopause Symptom Assessment Scale, PSY: psychological subscale of the Coronavirus 19 Phobia Scale, SOM: somatic subscale of the Coronavirus 19 Phobia Scale, SOC: social subscale of the Coronavirus 19 Phobia Scale, ECO: economic subscale of the Coronavirus 19 Phobia Scale.

Figure 1 provides the normal distribution of the subscale scores of the scales. When the descriptive characteristics of the women included in the study are examined, the mean age is 57.68 ± 8.60, and the mean body mass index (BMI) is 29.39 ± 5.01. Participants have been in menopause for an average of 9.81 ± 7.40 years. Overall, 81.4% of the participants are married, while 18.6% are single. Further, 21.1% of the participants are literate, and 51.6% have completed primary school. Regarding their thoughts on the menopausal period, 63.4% of the participants stated that they felt good, 29.2% felt bad, and 7.5% were undecided. The average number of births for participants is 3.86 ± 2.05, with 38% having experienced miscarriages.

In Table 1, the descriptive statistics for scale scores, results of normality tests, and reliability coefficients are provided. The mean scores for evaluating COVID-19 fear and menopausal symptoms fall within the moderate range. As indicated by the normality tests and histogram results (see Figure 1), all scales exhibit notable deviations from normality. Additionally, the scales demonstrate acceptable reliability, with Cronbach’s alpha values ranging from 0.631 to 0.921 [54]. Specifically, the assessment scales for COVID-19 fear and menopausal symptoms have shown high reliability levels of 0.863 and 0.914, respectively.

**Figure 2:**
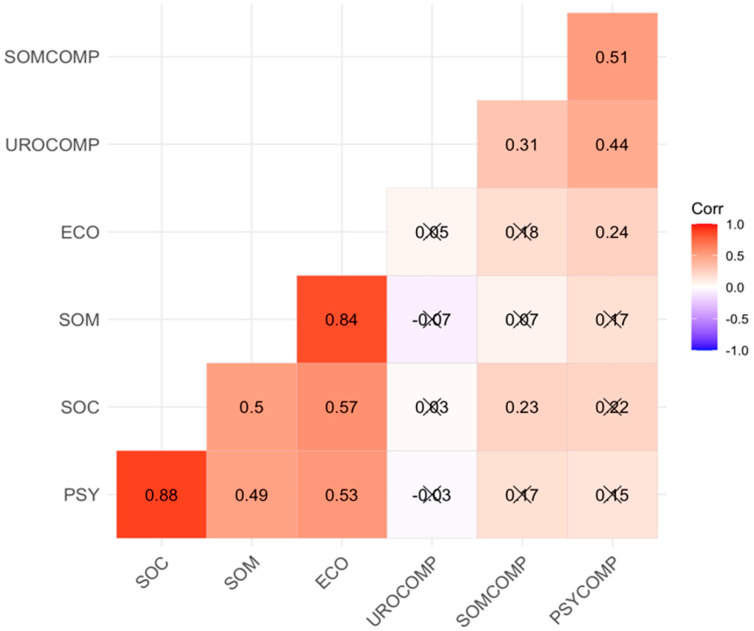
Correlation between subscales of COVID-19 fear scale and subscales of menopausal symptoms assessment scale. The symbol X points out the insignificance of the correlation coefficient.

In Figure 2, the correlation graph represents the relationships between scale scores. Based on the correlations, moderate- to high-level relationships are observed between the scale scores. There is a slight positive relationship between the social subscale of the COVID-19 fear scale and the somatic subscale of the menopausal symptoms scale. Similarly, a slight positive relationship is observed between the economic subscale of the COVID-19 scale and the psychological subscale of the menopausal symptoms scale.

There is no meaningful relationship between the economic, somatic, social, and psychological subscales of the COVID-19 fear scale and the urological subscale of the menopausal symptoms assessment scale (r = 0.05; r = −0.07; r = 0.03; r = −0.03).

There is no meaningful relationship between the economic, somatic, and psychological subscales of the COVID-19 fear scale and the somatic subscale of the menopausal symptoms assessment scale (r = 0.18; r = 0.07; r = 0.17).

There is no meaningful relationship between the somatic, social, and psychological subscales of the COVID-19 fear scale and the psychological subscale of the menopausal symptoms assessment scale (r = 0.17; r = 0.22; r = 0.15).

**Figure 3:**
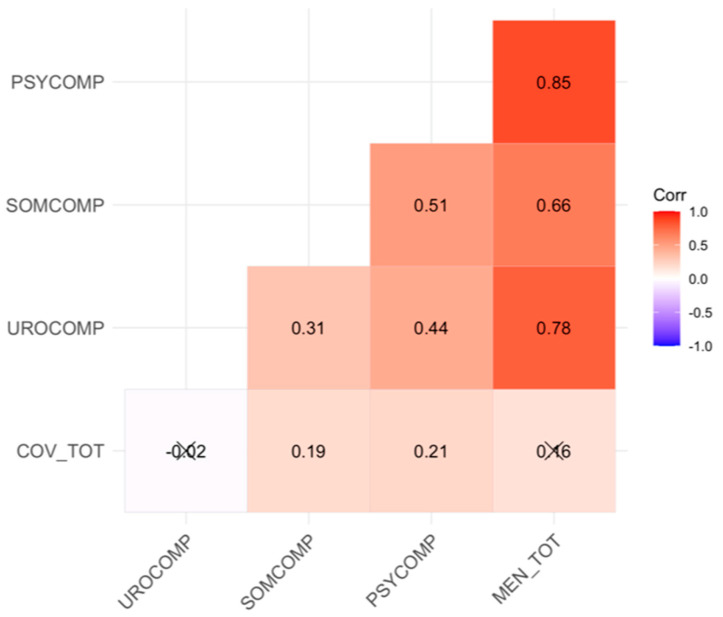
Correlation plot of the scale scores. The symbol X points out the insignificance of the correlation coefficient.

In Figure 3, the correlation graph depicts relationships between the total score averages of the scales. Based on the correlations, there is a slight positive relationship between the total score of the COVID-19 phobia scale and the somatic and psychological subscales of the menopausal symptoms assessment scale. However, there is no meaningful relationship between the total score of the COVID-19 phobia scale and the total score or the urological subscale of the menopausal symptoms assessment scale (r = 0.16; r = −0.02).

**Figure 4:**
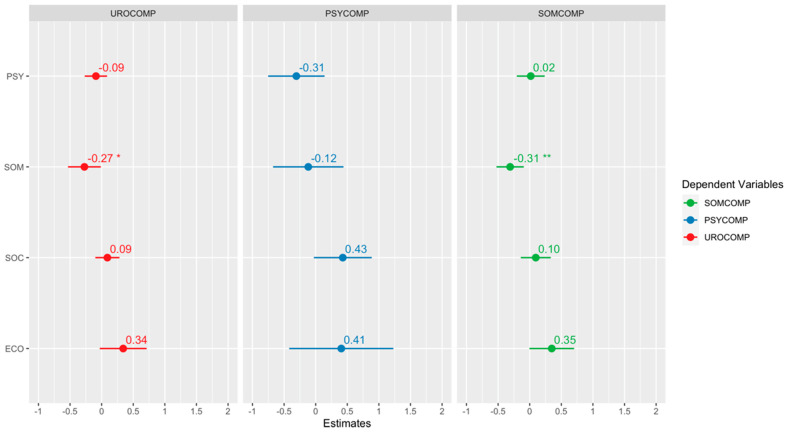
Regression results for the 25th percentile. “*” indicates a statistically significant effect of 5%, and “**” indicates a statistically significant effect of 1%.

**Figure 5:**
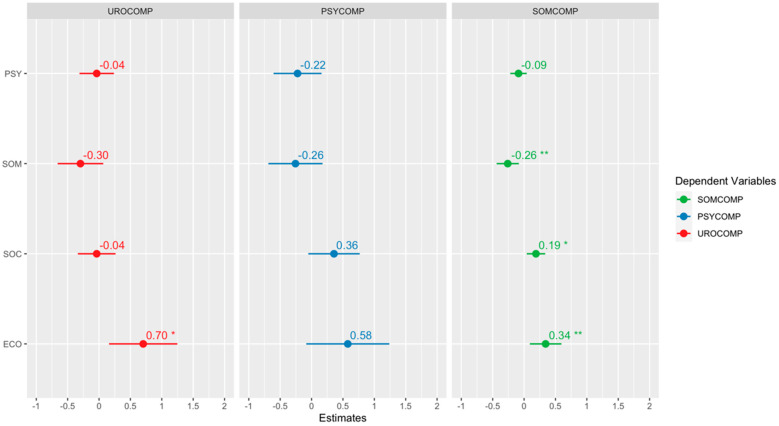
Regression results for the 50th percentile. “*” indicates a statistically significant effect of 5%, and “**” indicates a statistically significant effect of 1%.

**Figure 6:**
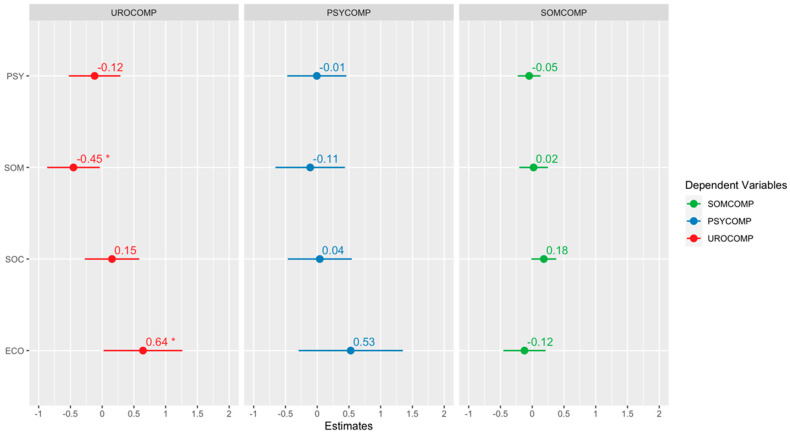
Regression results for the 75th percentile. “*” indicates a statistically significant effect of 5%.

Figure 4, Figure 5 and Figure 6 show the quantile regression model’s results and the beta coefficients’ graphical representations. The confidence interval of each coefficient shows its significance. If the interval contains zero, the corresponding coefficient is considered statistically insignificant. This allows us to inspect the impact of each independent variable visually. A star is added to the plot if a coefficient is statistically significant. Each plot includes the confidence intervals of the coefficients. If the confidence interval includes zero, the coefficient is considered insignificant. Additionally, the direction of the relationship is indicated by the sign of the coefficient: a positive coefficient indicates a positive relationship, while a negative coefficient indicates a negative relationship.

According to the regression analysis results, it was determined that the somatic subdimension of COVID-19 phobia had a statistically significant and negative effect on the evaluation of urological and somatic complaints related to menopause symptoms. Conversely, the economic factor of COVID-19 phobia was found to have a statistically significant and positive effect on urological and somatic complaints. Finally, it should be noted that the social factor of COVID-19 phobia has a statistically significant positive effect on somatic complaints.

## 4. Discussion

Our study is pioneering in its exploration of the link between fear of COVID-19 and menopausal symptoms among women in the menopausal period. A comprehensive review of the literature revealed a dearth of previous findings on this specific association. We believe our study can significantly bridge this gap in the literature. The results of our study underscore that the fear of COVID-19 among women in the menopausal period is indeed linked to the severity of specific menopausal symptoms.

Menopause is a physiologically normal phase in a woman’s life characterized by hormonal changes that lead to physical, psychological, and sexual symptoms. Studies indicate that women in menopause frequently seek healthcare, receive medical treatments, use alternative medicine, adopt diet and exercise regimens, and receive psychological and social support to manage these issues [55,56]. In our study, we observed that the social dimension of the fear of COVID-19 is associated with the somatic symptoms of menopause. This could be attributed to individuals in this period being exposed to quarantine during the COVID-19 pandemic, limiting their access to healthcare facilities, reducing their ability to engage in sufficient physical exercise, and hindering their social interactions. Quarantine measures during the pandemic forced individuals to change their daily routines, impacting their physical activity and exercise levels. Puccinelli et al. (2021) [57] found in their study that COVID-19 harmed physical activity, with less active individuals showing higher levels of mood disorders such as depression and anxiety.

In this study, we did not find a statistically significant relationship between the fear of COVID-19 and urological symptoms related to menopause. A probable reason for this could be that 81.4% of the patients included in the study were married, which may have contributed to their sexual function not being significantly affected. Similarly, Coronado et al. (2021) [58] concluded in their study during the pandemic that menopausal women who had an active sexual life tended to have a better quality of life. However, another study indicated that despite approximately 70% of women having partners, there was a trend of increasing sexual problems [59]. A cross-sectional study conducted in Iraq during the pandemic involving 296 women revealed that sexual function was significantly impacted by the stress brought about by COVID-19, adversely affecting women’s quality of life [60]. The differences in these findings may be attributed to variations in the prevalence and impact of the COVID-19 pandemic during the times when these studies were conducted.

It has been observed that the fear of COVID-19 contributes to an increase in somatic complaints among women in the menopausal period. Patients’ sleep problems have been reported to increase during the COVID-19 pandemic, and this situation is associated with both somatic and socioeconomic dimensions. In a study similar to ours during the COVID-19 pandemic, it was reported that the prevalence of severe sleep disorders increased and was associated with emotional and social loneliness [61]. Gökseven et al. investigated the levels of fear associated with COVID-19 in their study. They found that participants who reported sleep problems and expressed a need for psychological support had higher fear scores [62].

According to the results of this study, the economic dimension of fear related to COVID-19 is associated with psychological symptoms during menopause. During the pandemic, certain restrictions [63], increases in job losses, the negative economic impact of the pandemic [64], concerns about accessing various food and hygiene products [65,66], and stockpiling materials are believed to contribute to psychological stress in individuals. Economic anxieties are thought to lead to an increase in psychological symptoms [63].

In the present study, the results showed that a general fear of COVID-19 is associated with somatic and psychological symptoms during menopause. The emotion of fear is known to contribute to mood disorders such as anxiety, stress, and depression in individuals, and these emotional changes are thought to exacerbate physical complaints in menopausal women. According to a study by Alın (2022) [67], an increase in COVID-19 fear is correlated with an increase in psychological symptoms. Women in menopause, being aware that they are in a high-risk group for COVID-19 infection, may experience social isolation, fear, and psychological distress during this period. These factors can increase the severity of menopausal symptoms.

## 5. Conclusions

Our study’s findings are significant. We have concluded that fear of COVID-19 is linked to an increase in both somatic and psychological symptoms among women in the menopausal period. The various factors introduced by the pandemic, such as economic uncertainties, social isolation, and fear, have been observed to heighten the severity of these symptoms. These findings underscore the importance of understanding how health issues experienced by menopausal women are affected under pandemic conditions. They also highlight the crucial role of supportive treatment approaches during this period. The study concluded that COVID-19 fear is associated with an increase in both somatic and psychological symptoms among women in the menopausal period. The study concluded that COVID-19 fear is associated with an increase in both somatic and psychological symptoms among women in the menopausal period.

## Figures and Tables

**Table 1:** Descriptive findings of the scales.

Scale	Mean ± Standard Deviation	Min	Max	*p* (SW)	Alpha
MEN-TOT	22.10 ± 7.49	3	37	<0.001	0.914
SOMCOMP	4.97 ± 1.80	0	8	<0.001	0.631
PSYCOMP	11.54 ± 3.98	0	20	0.013	0.829
UROCOMP	5.59 ± 3.46	0	12	<0.001	0.812
COV-TOT	47.88 ± 13.44	19	82	<0.001	0.863
PSY	15.43 ± 4.96	5	25	0.009	0.921
SOM	10.10 ± 3.63	5	21	<0.001	0.911
SOC	14.55 ± 4.60	5	24	0.038	0.747
ECO	7.80 ± 2.49	4	16	<0.001	0.727

SD: standard deviation, Min: minimum, Max: maximum, SW: Shapiro–Wilk’s normality test, Alpha: Cronbach’s alpha reliability coefficient, MEN-TOT: total score of the Menopause Symptom Assessment Scale, COV-TOT: total score of the Coronavirus 19 Phobia Scale.

## Data Availability

The raw data supporting the conclusions of this article will be made available by the authors on request.

## References

[1] Minkin M.J. (2019). Menapause: Hormons, Lifestyle, and Optimizing Aging. Obstet. Gynecol. Clin. N. Am..

[2] Voedisch A.J., Dunsmoor-Su R., Kasirsky J. (2021). Menopause: A Global Perspective and Clinical Guide for Practice. Clin. Obstet. Gynecol..

[3] Santoro N., Roeca C., Peters B.A., Neal-Perry G. (2021). The MEnopause Transition: Signs, Symptoms, and Management Options. J. Clin. Endocrinol. Metab..

[4] WHO World Health Organization (2022). Menopause. https://www.who.int/news-room/fact-sheets/detail/menopause.

[5] Özcan H., Oskay Ü. (2013). Evidence based symptoms management in menopause. Göztepe Med. J..

[6] Taşkın L., Taşkın L. (2015). Stages of women’s life. Maternity and Women’s Health Nursing.

[7] Talaulikar V. (2022). Menopause Transition:Physiology and Symptoms. Best Pract. Res. Clin. Obstet. Gynaecol..

[8] Kaba F., Bozkurt Ö.D. (2020). Complementary and Alternatıve Therapıes in Menopause Symptoms. J. Midwifery Health Sci..

[9] Cowell A.C., Gilmour A., Atkinson D. (2024). Support Mechanisms for Women during Menopause: Perspectives from Social and Professional Structures. Women.

[10] Islam M.R., Gartoulla P., Bell R.J., Fradkin P., Davis S.R. (2015). Prevalence of Menopausal Symptoms in Asian Midlife Women: A Systematic Review. Climacteric.

[11] (2020). World Health Organization. Archived: WHO Timeline-COVID-19. https://www.who.int/news/item/27-04-2020-who-timeline---covid-19.

[12] Hui D.S., Azhar E.I., Madani T.A., Ntoumi F., Kock R., Dar O., Ippolito G., Mchugh T.D., Memish Z.A., Drosten C. (2020). The continuing 2019-nCoV epidemic threat of novel coronaviruses to global health—The latest 2019 novel coronavirus outbreak in Wuhan, China. Int. J. Infect. Dis..

[13] Arpaci I., Karataş K., Baloğlu M. (2020). The development and initial tests for the psychometric properties of the COVID-19 Phobia Scale (C19P-S). Personal. Individ. Differ..

[14] Biçer İ., Çakmak C., Demir H., Kurt M.E. (2020). Coronavirus anxiety scale short form: Turkish validity and reliability study. Anatol. Clin..

[15] Erkin Ö., Konakçı G., Duran S. (2021). Secondary traumatic stress in nurses working with patients with suspected/confirmed COVID-19 in Turkey. Perspect. Psychiatr. Care.

[16] An Y., Yang Y., Wang A., Li Y., Zhang Q., Cheung T., Ungvari G.S., Qin M.Z., An F.R., Xiang Y.T. (2020). Prevalence of depression and its impact on quality of life among frontline nurses in emergency departments during the COVID-19 outbreak. J. Affect. Disord..

[17] Arnetz J.E., Goetz C.M., Sudan S., Arble E., Janisse J., Arnetz B.B. (2020). Personal protective equipment and mental health symptoms among nurses during the COVID-19 pandemic. J. Occup. Environ. Med..

[18] Zhu Z., Xu S., Wang H., Liu Z., Wu J., Li G., Miao J., Zhang C., Yang Y., Sun W. (2020). COVID-19 in Wuhan: Sociodemographic characteristics and hospital support measures associated with the immediate psychological impact on healthcare workers. EClinicalMedicine.

[19] Ibrahim N.K. (2016). Zika virus: Epidemiology, current phobia and preparedness for upcoming mass gatherings, with examples from World Olympics and pilgrimage. Pak. J. Med. Sci..

[20] Kim C.W., Song H.R. (2017). Structural relationships among public’s risk characteristics, trust, risk perception and preventive behavioral intention: The case of MERS in Korea. Crisisnomy.

[21] Liu Z.G., Zhang K.R., Lu Z.X. (2005). Follow-up study on phobia emotion of SARS patients. J. Shanxi Med. Univ..

[22] Tausczik Y., Faasse K., Pennebaker J.W., Petrie K.J. (2012). Public anxiety and information seeking following the H1N1 outbreak: Blogs, newspaper articles, and Wikipedia visits. Health Commun..

[23] Theresa N.C., Christian N.G., Nnadi F.U. (2014). The pervasiveness of Ebola virus disease in Africa: Implication for economy, ecology and socio-religious dynamics. Int. J. Hum. Sci..

[24] Nacar G., Türkmen S., Sinen R., Taşhan S.T. (2022). The effect of COVID-19 on sleep state in postmenopausal women. Karya J. Health Sci..

[25] Dehghani M., Hakimi H., Talebi M., Rezaee H., Mousazadeh N., Ahmadinia H., Almasi S. (2023). The relationship between fear of COVID-19 and obsessive-compulsive disorder. BMC Psychol..

[26] Bryant C., Judd F.K., Hickey M. (2012). Anxiety during the menopausal transition: A systematic review. J. Affect. Disord..

[27] Gracia C.R., Freeman E.W. (2018). Onset of the Menopause Transition: The Earliest Signs and Symptoms. Obstet. Gynecol. Clin. N. Am..

[28] Tangen T., Mykletun A. (2008). Depression and anxiety through the climacteric period: An epidemiological study (HUNT II). J. Psychosom. Obstet. Gynaecol..

[29] Tang R.Y., Luo M., Fan Y.B., Xie Z.L., Huang F.L., Zhang D.D., Liu G.F., Wang Y.P., Lin S.Q., Chen R. (2022). Effects of Menopause on Depressive and Anxiety Symptoms in Community Women in Beijing. Zhonghua Fu Chan Ke Za Zhi.

[30] Alblooshi S., Taylor M., Gill N. (2023). Does menopause elevate the risk for developing depression and anxiety? Results from a systematic review. Australas. Psychiatry.

[31] Rahul P., Pritamkumar B. (2017). Study of menopause complains in relation with trait anxiety and personality traits. Indian J. Health Wellbeing.

[32] Salehi L., Rahimzareh M., Molaei E., Zaheri H., Esmaelzadeh-Saeieh S. (2020). The Relationship Among Fear and Anxiety of COVID-19, pregnancy experience, and Mental Health Disorder in Pregnant Women: A Structural Equation Model. Brain Behav..

[33] Luong T.C., Pham T.T., Nguyen M.H., Do A.Q., Pham L.V., Nguyen H.C., Nguyen H.C., Ha T.H., Dao H.K., Trinh M.V. (2021). Fear, anxiety and depression among pregnant women during COVID-19 pandemic: Impacts of healthy eating behaviour and health literacy. Ann. Med..

[34] Naghizadeh S., Mirghafourvand M. (2021). Relationship of fear of COVID-19 and Pregnancy-related Quality of life during the COVID-19 pandemic. Arch. Psychiatr. Nurs..

[35] Güvenç G., Yeşilçınar İ., Özkeçeci F., Öksüz E., Özkeçeci C.F., Konukbay D., Kök G., Karaşahin K.E. (2021). Anxiety, depression, and knowledge level in postpartum women during the COVID-19 pandemic. Perspect. Psychiatr. Care.

[36] Fan H.S.L., Choi E.P.H., Ko R.W.T., Kwok J.Y.Y., Wong J.Y.H., Fong D.Y.T., Shek N.W.M., Ngan H.Y.S., Li J., Huang Y. (2022). COVID-19 related fear and depression of pregnant women and new mothers. Public Health Nurs..

[37] Aydın R., Bostan F.S., Kabukcuoğlu K. (2022). Two wars on one front: Experiences of gynaecological cancer patients in the COVID-19 pandemic. Eur. J. Cancer Care.

[38] Kiyak S., Türkben Polat H. (2022). The relationship between death anxiety and COVID-19 fear and anxiety in women with breast cancer. OMEGA-J. Death Dying.

[39] Uslu-Sahan F., Yesilcınar I., Kurt G., Hancer E., Guvenc G. (2023). Effects of COVID-19 fear and anxiety on attitudes towards complementary and alternative medicine use in women with gynecological cancer during the COVID-19 pandemic. J. Integr. Med..

[40] Hazar S., Güleç Şatir D. (2023). The effect of fear of COVID-19 on health-seeking behaviors and Pap smear test rates in women. Women Health.

[41] Calpbinici P., Uzunkaya Öztoprak P. (2023). The Effect of Fear of COVID-19 on Women’s Attitudes toward Cancer Screening and Healthy Lifestyle Behaviors: A Cross-Sectional Study. Indian J. Gynecol. Oncol..

[42] Scheineder H.P.G., Heinemann L.A.J., Rosemeier H.P., Potthoff P., Behre H.M. (2000). The Menapouse Rating Scale (MRS) Reliability of scores of menapousal complaints. Climacteric.

[43] Scheineder H.P.G., Heinemann L.A.J., Rosemeier H.P., Potthoff P., Behre H.M. (2000). The Menapouse Rating Scale (MRS) Comparison with Kupperman index and Quality of Life Scale SF-36. Climacteric.

[44] Heinemann K., Ruebig A., Potthoff P., Schneider H.P., Strelow F., Heinemann L.A., Do M.T. (2004). The Menopause Rating Scale (MRS) scale: A methodological review. Health Qual. Life Outcomes.

[45] Potthoff P., Heinemann L.A., Schneider H.P., Rosemeier H.P., Hauser G. (2000). The menopause Rating Scale (MRSII): Methodological Standardization in the German Population. Zentralbl. Gynakol..

[46] Gürkan C.Ö. (2005). The validity and reliability of Turkish version of menopouase rating scale. Nurs. Forum.

[47] Kaveh V. (2018). DetMCD: Implementation of the DetMCD Algorithm (Robust and Deterministic Estimation of Location and Scatter). R Package Version 0.0.5. https://CRAN.R-project.org/package=DetMCD.

[48] Revelle W. (2022). Psych: Procedures for Personality and Psychological Research. Northwestern University, Evanston, Illinois, USA. Version 2.2.9. https://CRAN.R-project.org/package=psych.

[49] Wickham H. (2016). ggplot2: Elegant Graphics for Data Analysis.

[50] R Core Team (2023). R: A Language and Environment for Statistical Computing.

[51] Kassambara A. (2023). ggcorrplot: Visualization of a Correlation Matrix using ‘ggplot2’. R Package Version 0.1.4.1. https://CRAN.R-project.org/package=ggcorrplot.

[52] Marrie R.A., Dawson N.V., Garland A. (2009). Quantile regression and restricted cubic splines are useful for exploring relationships between continuous variables. J. Clin. Epidemiol..

[53] Lüdecke D. (2023). sjPlot: Data Visualization for Statistics in Social Science. R Package Version 2.8.15. https://CRAN.R-project.org/package=sjPlot.

[54] Ursachi G., Horodnic I.A., Zait A. (2015). How reliable are measurement scales? External factors with indirect influence on reliability estimators. Procedia Econ. Financ..

[55] Matsuzaki K., Fukuoka M., Uemura H., Yasui T. (2018). Differences in strategies for coping with menopausal symptoms in full-time workers and part-time workers in Japan. Int. J. Nurs. Midwifery.

[56] İkiışık H., Turan G., Kutay F., Karamanlı D.C., Gülen E., Özdemir E., Taşdemir E., Maral I. (2020). Awareness of menopause and strategies to cope with menopausal symptoms of the women aged between 40 and 65 who consulted to a tertiary care hospital. ESTÜDAM Halk Sağlığı Dergisi.

[57] Puccinelli P.J., da Costa T.S., Seffrin A., de Lira C.A.B., Vancini R.L., Nikolaidis P.T., Knechtle B., Rosemann T., Hill L., Andrade M.S. (2021). Reduced level of physical activity during COVID-19 pandemic is associated with depression and anxiety levels: An internet-based survey. BMC Public Health.

[58] Coronado P.J., Fasero M., Otero B., Sanchez S., De la Viuda E., Ramirez-Polo I., Llaneza P., Mendoza N., Baquedano L. (2021). Health-related quality of life and resilience in peri-and postmenopausal women during COVID-19 confinement. Maturitas.

[59] Serra C.O., Leite P.M.G., Bezerra A.B., Freitas L., Veras L., Costa M.D., Gonçalves L.L.C., dos Santos Maciel L.Y. (2022). Comparison of climacteric symptoms, quality of life, and self-care attitudes before and during the COVID-19 pandemic. J. Menopausal Med..

[60] Daneshfar Z., Jahanian Sadatmahalleh S., Youseflu S., Bahri Khomami M., Kazemnejad A. (2021). Influential factors on quality of life in married Iranian women during the COVID-19 pandemic in 2020: A path analysis. BMC Women’s Health.

[61] Huang Y., Fietze I., Penzel T. (2022). Analysis of the correlations between insomnia and mental health during the COVID-19 pandemic in Germany. Somnologie.

[62] Gokseven Y., Ozturk G.Z., Karadeniz E., Sarı E., Tas B.G., Ozdemir H.M. (2022). The fear of COVID-19 infection in older people. J. Geriatr. Psychiatry Neurol..

[63] Cakır Kardeş V. (2020). Mental and Behavioral Evaluation of During and After the Pandemic. Turk. Diyab. Obez..

[64] Soylu Ö.B. (2020). The sectoral effects of COVID-19 in Turkish economy. Eurasian J. Soc. Econ. Res..

[65] İnce M., Kadıoğlu Tor C. (2020). Effect of Consumers Increasing Stocking Request with the COVİD-19 (Corona) Virus on Online Purchasing Behavior. Int. J. Soc. Res..

[66] Eşiyok A., Uslu Divanoğlu S. (2022). The Effect of COVİD-19 Pandemic on Consumer Purchase Behaviour. ASED.

[67] Alın T. (2022). Examining the Relationship between Fear of COVID-19 and Psychological Symptoms. Master’s Thesis.

